# Association between the ERCC1 polymorphism and platinum-based chemotherapy effectiveness in ovarian cancer: a meta-analysis

**DOI:** 10.1186/s12905-017-0393-z

**Published:** 2017-06-17

**Authors:** Ning Tang, Dan Lyu, Yan Zhang, Haiping Liu

**Affiliations:** 1 Reproductive Medicine Center, Jinan Military General Hospital, 25 Shifan Road, Tianqiao District, Jinan, Shandong Province 250031 China; 2 Department of Pain, Tianjin First Center Hospital, Nankai District, Tianjin, 300192 China

**Keywords:** ERCC1 C19007T, Platinum-based chemotherapy, Ovarian cancer

## Abstract

**Background:**

Ovarian cancer is a prominent public health problem which affects people all around the world. Platinum-based chemotherapy is a common treatment for ovarian cancer, however, the effectiveness of chemotherapy varies from patient to patient. The excision repair cross complementation group 1 (ERCC1) protein may mediate chemotherapy resistance. A meta-analysis was conducted to explore whether platinum-based chemotherapy effectiveness could be attributed to the ERCC1 C19007T polymorphisms.

**Methods:**

Seven major databases (EMBASE, Web of Science, Pubmed, Springer Link, Chinese National Knowledge Infrastructure (CNKI), EBSCO and Science Direct databases) were searched for eligible studies. Crude odds ratios (ORs) with 95% confidence intervals (CIs) were calculated to evaluate the results.

**Results:**

In this meta-analysis, 1169 subjects (425 non-responders and 744 responders) from 8 studies were included. The overall OR (C vs. T alleles) using random model was 1.07 (95% CI 0.75-1.52, *P* = 0.7), which was not statistically significant. Moreover, there was no significant difference in the analysis by race.

**Conclusion:**

There is no association between the ERCC1 C19007T polymorphism and platinum-based chemotherapy effectiveness in ovarian cancer. The polymorphism did not have a significant impact on platinum-based chemotherapy in non-responders and responders.

## Background

World Health Organization (WHO) reports that ovarian cancer accounts for 190,000 new cases annually [1] and is the leading cause of death among the gynecologic cancers [2]. Platinum-based chemotherapy drugs are first-line treatments for ovarian cancer [3]. However, a large number of patients do not respond to platinum-based chemotherapy due to drug resistance. Previous research shows that the nucleotide excision repair (NER) system plays an important role in platinum resistance to chemotherapy [4]. It repairs platinum-induced DNA damage by removing the damaged fragments in the DNA molecule, and then synthesizing DNA by DNA polymerase. ERCC1 (excision repair cross complementation group 1) is a key gene involved in NER. The ERCC1 mRNA and protein expressions are associated with the effectiveness of platinum-based chemotherapy in several cancers [5]. Studies have indicated that ERCC1 mRNA and protein expressions can be used as biomarkers for patients’ outcomes in ovarian cancer [6, 7]. Moreover, the single nucleotide polymorphisms (SNPs) of ERCC1 are considered as potential predictive biomarkers for many different types of cancers [8, 9]. However, the sample sizes of these SNPs studies are small. Some studies on the efficacy of platinum-induced therapy have shown inconsistent results [10, 11]. Moxley [10] reported that the C19007T SNP (rs11615) in ERCC1 (codon-118) was not associated with the response to platinum treatment in patients with epithelial ovarian cancer (EOC). On the other hand, Qi [11] reported that CC, CT and TT frequency in rs11615 was significantly different between responders and non-responders to platinum treatment in patients with EOC. Therefore, a comprehensive review of rs11615 in the responders and non-responders of EOC patients is required. In this study, we performed a meta-analysis of the published literature to assess the association between the ERCC1 protein expression and the response to platinum-based chemotherapy in patients with ovarian cancer.

## Methods

### Literature review

**Fig. 1:**
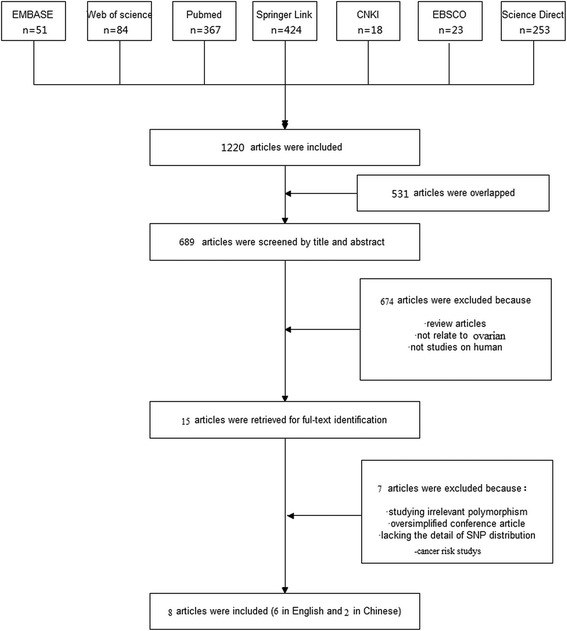
A flow chart showing the study selection procedure. CNKI: China National Knowledge Infrastructure

We searched the EMBASE, Web of Science, PubMed, Springer Link, Chinese National Knowledge Infrastructure (CNKI), EBSCO and Science Direct databases to identify the publications that reported the association between the excision repair cross-complementation group 1 polymorphism and platinum-based chemotherapy effectiveness in ovarian cancer from March, 1980 to December, 2014. The key words for chemotherapy effectiveness, including outcome, response, resistance, etc. were complex; therefore, we used the keywords as follows: ‘ovarian cancer, excision repair cross-complementation group 1, polymorphism’, ‘ovarian cancer, excision repair cross-complementation group 1, SNP’. ‘ovarian cancer, excision repair cross-complementation group 1, allele’, ‘ovarian cancer, ERCC1, polymorphism’, ‘ovarian cancer, ERCC1, SNP’, ‘ovarian cancer, ERCC1, allele’, ‘ovarian carcinoma, excision repair cross-complementation group 1, polymorphism’, ‘ovarian carcinoma, excision repair cross-complementation group 1, SNP’, ‘ovarian carcinoma, excision repair cross-complementation group 1, allele’, ‘ovarian carcinoma, ERCC1, polymorphism’, ‘ovarian carcinoma, ERCC1, SNP’, ‘ovarian carcinoma, ERCC1, allele’. The articles related to platinum-based chemotherapy effectiveness were manually extracted from the articles related to ovarian cancer therapy or other chemotherapy effectiveness. Responder and non-responder were chosen as the parameter in this study. Responder was defined as “complete response (disappearance of tumor, which was confirmed at 4 weeks) + partial response (30% decrease in tumor size, which was confirmed at 4 weeks). Stable disease or progressive disease was not included. Non-responder was defined as disease progression during platinum-based chemotherapy or recurrence during therapy or without completing the therapy. After excluding duplicates, titles and abstracts were reviewed. Studies were included if they: 1) were non-responder-responder studies, which comparing the difference between the non-responders and responders to the platinum-based chemotherapy in EOC; 2) reported genotype distribution in both non-responders and responders; 3) articles in English or Chinese with an English abstract. The studies were excluded if they were: 1) review articles; 2) not related to ovarian cancer; 3) animal or in vitro study; 4) not related to the association between ovarian cancer and polymorphism; 5) not related to ERCC1. The study selection process was summarized in Fig. 1.

### Data extraction

For each study, the following information was extracted from the original articles: the name of the first author, the year of publication, country of origin, race of study population, genotype distributions for each polymorphism in both cases and controls, statistical characteristics of each study (sample size, gender and age distribution of cases and controls, *P* values for HWE evaluation), source of controls, and genotyping methods (Table 1).

**Table 1:** Characteristics of the Studies Included in the Analysis

Authors	Year of publication	Host ethnicity	Age (years Mean ± SD)		Samples (n)		Regimen of chemotherapy	Genotyping method
			Resistant	Responder	Resistant	Responder		
Sokbom K. et al. [18]	2009	Asian	No data	No data	20	40	Atinum-Taxane	PCR-RFLP
Steffensen K.D. et al. [20]	2011	Caucasian	54.92 ± 11.42	52.33 ± 10.75	155	144	Carboplatin and Paclitaxel	PCR-DNA chip
Smith S. et al. [16]	2007	Caucasian	66.9 ± 7.8	68.33 ± 9.5	48	128	carboplatin or cisplatin and paclitaxel	PCR-RFLP
Qi B. et al. [11]	2013	Asian	No data	No data	73	147	Platium- Based, did not provide specific information	PCR-RFLP
Bösmüller H. et al. [19]	2011	Caucasian	59.7 ± 18.3	62.33 ± 13.5	14	27	Carboplatin-Taxane	PCR-RFLP
Steffensen K.D. et al. [17]	2008	Caucasian	61.1 ± 20	68.4 ± 14.7	12	88	Platium- Based, did not provide specific information	PCR-DNA chip
Moxley K.M. et al. [10]	2013	Caucasian	64.89 ± 13.2	71.28 ± 12.9	32	32	Platium- Based Chemotherapy	PCR-RFLP
Yang S. [21]	2011	Asian	76.9 ± 17.6	72.9 ± 15.9	71	138	Cisplatin or Carboplatin and cyclophosphamide	PCR-RFLP

*PCR* polymerase chain reaction, *RFLP* restriction fragment length polymorphism

### Statistical analyses

Statistical analyses were conducted with Stata 13.0 (College Station, TX). Hardy-Weinberg Equilibrium (HWE) was performed in controls by asymptotic Pearson’s Chi-square test for each polymorphism in each study. The association between polymorphism and platinum-based chemotherapy effectiveness in ovarian cancer was estimated with odds ratios (OR) and corresponding 95% confidence intervals (CIs). Between studies heterogeneity was tested using Q test and I2 test, and the heterogeneity was considered significant if *P*-value was less than 0.05. Fixed-effects model was adopted when *P*-value was more than 0.05; otherwise random-effects model was used [12]. The publication bias was evaluated using Begg’s test, Egger’s test and Harbord’s test [13–15]. *P* < 0.05 was considered statistically significant.

## Results

### The characteristics of included studies

**Fig. 2:**
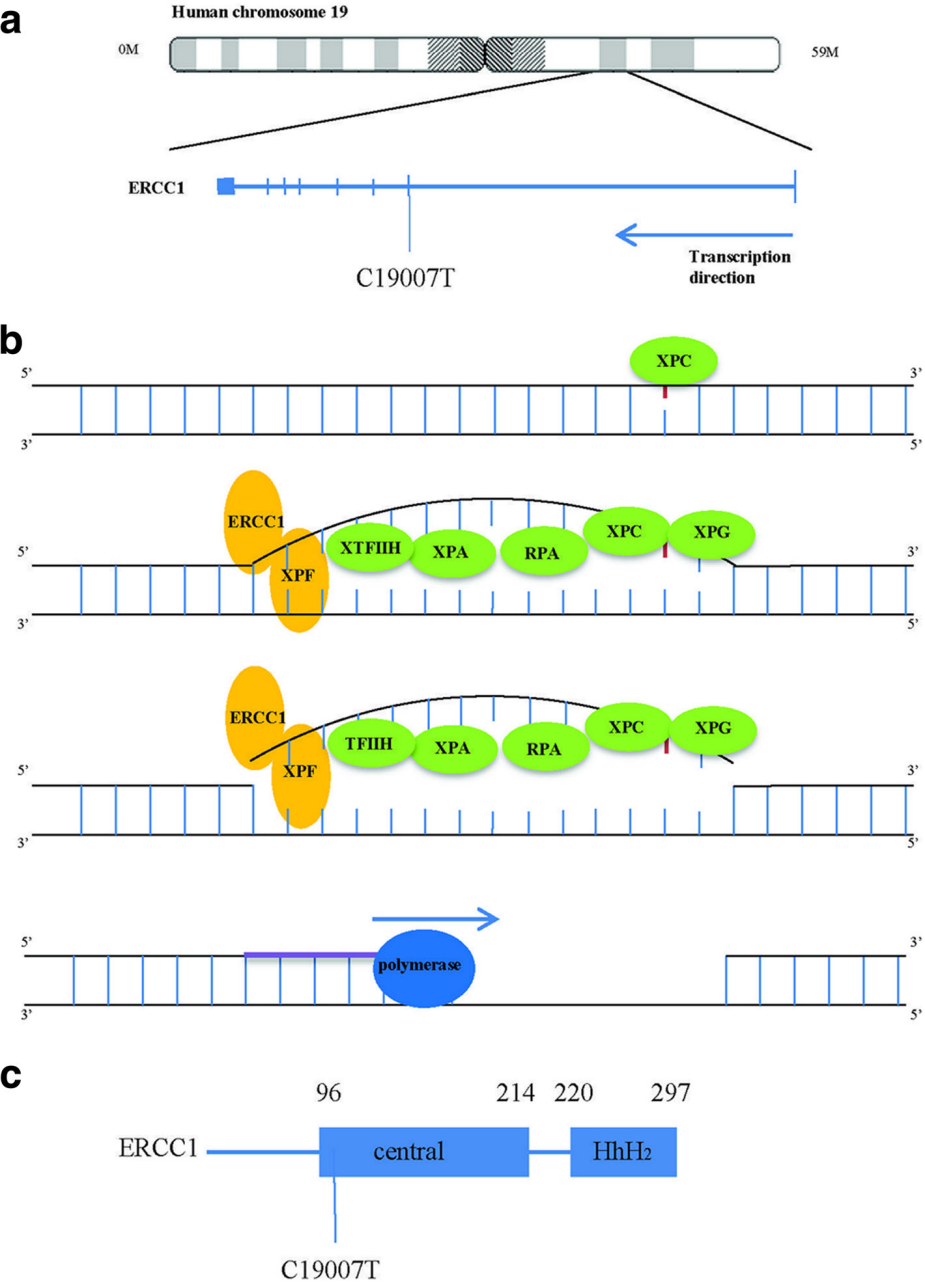
The location, function and structure of ERCC1. **a** The location of RECC1. ERCC1, excision repair cross complementation group 1. **b** The function of ERCC1. XPC binds to DNA damage site (*red circle*), XPA, XPG, RPA and TFIIH attach to the distorted DNA and then nucleases make the incisions on the damaged strand. ERCC1–XPF complex cuts 5′ of the lesion, whereas XPG cuts 3′. The excised DNA is replaced in a DNA-repair synthesis reaction that is catalyzed by DNA polymerase. XPA: Xeroderma pigmentosum complementation group A; XPC: Xeroderma pigmentosum complementation group C; XPF: Xeroderma pigmentosum complementation group F; XPG: Xeroderma pigmentosum complementation group G; RPA: Replication protein A; TFIIH: Transcription factor II Human; **c** The structure of ERCC1. ERCC1 contains a central domain (from 96 amino acid residue to 214 amino acid residue) and an HhH_2_ domain (from 240 amino acid residue to 297 amino acid residue). The HhH_2_ domain is linked to XPF, whereas the central domain combines with DNA. HhH_2_, helix-hairpin-helix domain

A total of 1220 articles were obtained from the literature search. As shown in Fig. 1, after excluding the duplicates, 689 abstracts were reviewed. A total of 15 research articles reporting the association of human ERCC1 gene polymorphisms and platinum-based chemotherapy effectiveness in ovarian cancer were identified. Seven articles were excluded after reviewing the full-text article because the polymorphism they studied didn’t appear in other articles, or they did not study chemotherapy effectiveness or they did not include the SNP distribution details. Finally, 8 studies from 8 articles were included in this analysis [10, 11, 16–21]. Six articles were published in English and two articles were published in Chinese. The SNP C19007T (rs11615) was studied. As shown in Table 1, the race of the study population included Asian and Caucasian. The pooled study population consisted of 1169 subjects (425 non-responders and 744 responders). For example, Steffensen et al. [20] showed that 13 patients did not respond to the first line of treatment while 155 patients relapsed during the long-term treatment. Therefore, we chose the 155 patients as our subjects. The genotype and allele distributions of all polymorphisms were shown in Table 2. These genotype distributions in controls deviated from HWE. The location of each SNP was shown in Fig. 2 based on information from CNBI SNPs database.

**Table 2:** Genotype and Allele Distribution of ERCC1 Polymorphisms in ovarian cancer Resistant and Responders

SNP	Study	Resistant					Responder					Resistant	Responder	HWE	
		C/C	C/T	T/T	C	T	C/C	C/T	T/T	C	T	Number (%)	Number (%)	Chi	*p*
ERCC1 C19007T	Sokbom K. et al. [18]	15	5	0	35	5	20	16	4	56	24	20 (50)	40 (50)	0.09	0.76
	Steffensen K.D. et al. [20]	16	72	67	104	206	15	71	58	101	187	155 (52)	144 (48)	0.98	0.32
	Smith S. et al. [16]	11	22	15	44	52	23	60	45	106	150	48 (27)	128 (73)	0.15	0.71
	Qi B. et al. [11]	38	26	9	102	44	78	67	2	223	71	73 (33)	147 (67)	8.76	0.003
	Bösmüller H. et al. [19]	6	5	3	17	11	7	9	11	23	31	14 (34)	27 (66)	2.74	0.1
	Steffensen K.D. et al. [17]	3	7	2	13	11	12	34	42	58	118	12 (12)	88 (88)	1.39	0.24
	Moxley K.M. et al. [10]	10	13	9	33	31	14	8	10	36	28	32 (50)	32 (50)	8.04	0.005
	Yang S. [21]	28	31	12	87	55	70	67	1	207	69	71 (34)	138 (66)	11.98	0.0005

*ERCC1* excision repair cross complementation group 1, *HWE* Hardy-Weinberg Equilibrium

### Data synthesis by polymorphism

#### ERCC1 C19007T polymorphism

**Fig. 3:**
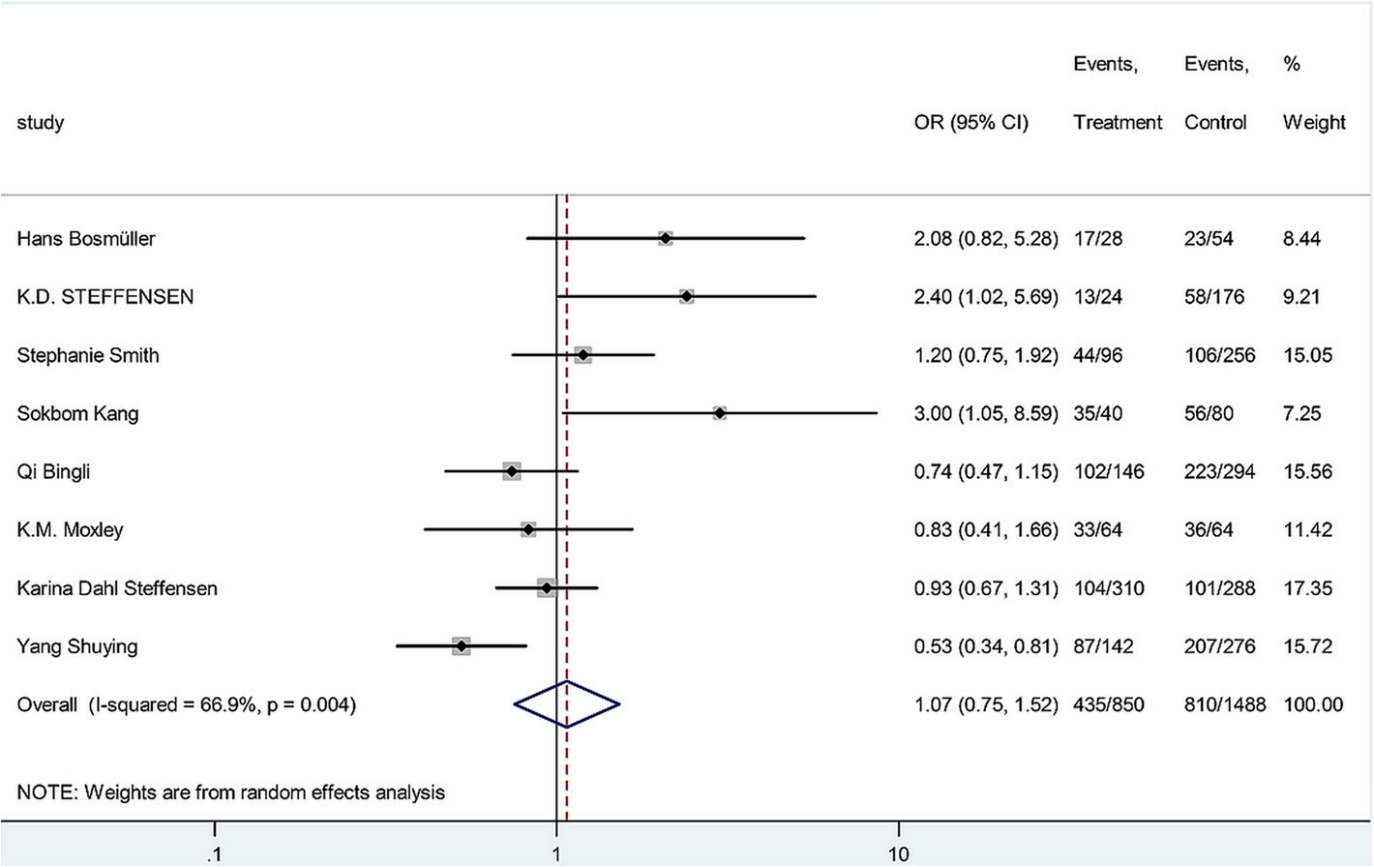
Forrest plot of the association between C19007T and treatment response in allele comparison (C vs. T)

Eight case–control studies (425 non-responders and 744 responders) addressed the relationship between C19007T polymorphism and the effectiveness of chemotherapy in ovarian cancer. As shown in Table 3, the heterogeneity was significant (P = s 0.004, I^2^ = 66.9%). The overall OR (C vs. T alleles) using random model was 1.07 (95% CI 0.75-1.52), *P* = 0.7. We also performed analysis using other models, but we did not identify any association (Table 3). The results show that there is no association between C19007T and platinum-based chemotherapy effectiveness in ovarian cancer. Subgroup analysis in allele comparison (C vs. T) was performed by race (Fig. 3). The race of the subjects in the studies from Steffensen et al. [17, 20], Bösmüller et al. [19], and Moxley et al. [10] was Caucasian; whereas the race of the subjects in the studies from Sokbom K et al. [18], Qi B et al. [11], and Yang et al. [21] was Asian. Furthermore, the same ethnicity (Chinese, studied by Qi B et al. [11] and Yang Shuying et al. [21]) or the same author (Steffensen et al. [17, 20]) showed different outcomes.

**Table 3:** Meta-analysis by Genetic Models for C19007T Polymorphisms

SNP	Genetic model	Participants (%)	OR (95% CI)	Z	*P* value	I^2^%	P_het_	Effect model	Begg’s test *p* > │z│	Egger’s test *p* > │t│	Harbord test *p* > │t│
ERCC1 C19007T	CC vs. CT + TT	1169 (50)	1.0 (0.75, 1.33)	0	0.99	26.6	0.22	Fixed	0.08	0.06	0.07
	TT vs. CT + CC	1169 (50)	1.16 (0.53, 2.55)	0.38	0.7	72.5	0.001	Random	0.81	0.83	0.91
	CC vs. CT	879 (39)	1.10 (0.81, 1.49)	0.59	0.56	0.0	0.66	Fixed	0.62	0.72	0.75
	CC vs. TT	656 (28)	0.85 (0.33, 2.22)	0.33	0.74	72.4	0.001	Random	0.81	0.86	0.56
	C vs. T	2338 (100)	1.07 (0.75, 1.52)	0.38	0.7	66.9	0.004	Random	0.03	0.06	0.07

*ERCC1* excision repair cross complementation group 1

### Publication bias

**Fig. 4:**
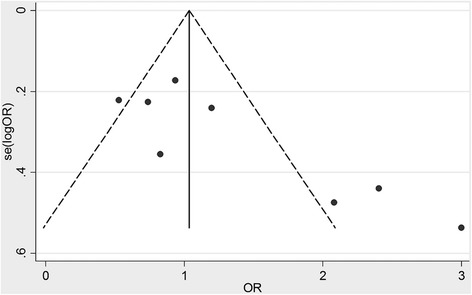
Funnel plot of meta-analysis according to Begg’s test

Begg’s test, Egger’s test, Harbord’s test and Begg’s funnel plot were calculated or plotted to detect the publication bias of the meta-analysis. The shapes of the funnel plot for the ERCC1 C19007T polymorphism demonstrated an asymmetrical result in C vs. T model (Fig. 4). Nevertheless, Egger’s test (*P* = 0.06) and Harbord’s test (*P* = 0.07) indicated that the C vs. T model was not significantly different (Table 3). The publication bias was inconsistent using different tests. In a previous study, Begg’s method had a stronger statistical and discriminatory power [22].

## Discussion

Platinum-based therapy is used in the treatment of several human cancers by damaging DNA in cancer cells at interphase. The NER pathway plays an important role in the repair of DNA damage [23, 24]. It has been reported that a number of single nucleotide polymorphisms (SNPs) of NER genes can alter the function of the respective genes, which might contribute to inter-individual variations of DNA repair capacity [6, 25]. Previous results suggest that DNA repair capacity might be influenced by genetic polymorphisms [26, 27]. The C19007T is a common polymorphism of ERCC1 gene and is associated with the effectiveness of platinum-based treatment in several types of cancers [28, 29]. Moxley et al. evaluated the association of ERCC1 and the response to platinum-based chemotherapy in ovarian cancer and found that negative ERCC1 expression was associated with a better response to platinum-based chemotherapy [10]. Their results proved that genetic variation in ERCC1 might contribute to impaired DNA repair capacity [10].

NER is the main mechanism by which platinum-induced damage is removed from DNA in mammalian cells. The recognition and incision step of the NER reaction requires the XPC (Xeroderma pigmentosum complementation group C), XPA (Xeroderma pigmentosum complementation group A), RPA (Replication protein A), TFIIH (Transcription factor II Human), XPG (Xeroderma pigmentosum complementation group G) and the ERCC1–XPF (Xeroderma pigmentosum complementation group F) complex. XPC binds to the DNA, which alters its structure and allows the access of other repair factors. XPA, RPA, XPG and TFIIH attach to the distorted DNA and then the nucleases makes incisions on the damaged strand. ERCC1–XPF complex cuts 5′ of the lesion, whereas XPG cuts 3′. Then the excised DNA is replaced by a DNA-repair synthesis reaction that is catalyzed by DNA polymerase (Fig. 2b) [30].

The C19007T SNP is located on the exon of ERCC1 gene on chromosome 19q13.32 (Fig. 2a) [31]. It codes 118 Asparagine (Asn). The mutation does not change the amino acid. The ERCC1 protein has two main functional domains: a central domain and a helix-hairpin-helix (HhH_2_) domain (Fig. 2c). The central domain binds to DNA strand, whereas ERCC1 and XPF form heterodimer by HhH2 domain [32]. C19007T allele is located in the central domain. In a previous study, this allele was shown not to affect gene expression, but thought to possibly be a “passenger” that affects other polymorphisms and has a strong linkage disequilibrium with C19007CT, especially in Caucasians [32].

A total of 8 eligible studies, including 1169 subjects (425 non-responders and 744 responders) were identified and analyzed in this meta-analysis study. We found that C19007T polymorphism is not associated with the outcome of platinum-based chemotherapy in ovarian cancer using models comparison. However, an asymmetric funnel plot indicated the conflicting results of race-based comparisons and the legacy of the current study. In the subgroup analysis by race, a significant association was detected in ovarian cancer in homozygote and recessive models in our study. In terms of stratified analysis by race, we found this polymorphism had a trend of an increased treatment response in Asian populations in homozygote and recessive models comparisons. On the other hand, the studies of Caucasian population show conflicting results. For example, Steffensen [20] and Bösmüller [19] indicated that the OR was greater than 1, whereas, Smith [16], Steffensen [17] and Moxley [10] did not show significant difference. Moreover, even the same author, Steffensen K.D., had conflicting results in 2011 [20] and 2008 [17]. It suggests that the sample sizes of this study should be expanded. Compared with the other studies, the article of Steffensen K.D in 2011 may be more reliable because of the larger sample size (199 vs. 100).

This meta-analysis has several limitations. Firstly, detailed individual data are not available, and a more precise analysis should be conducted on other covariates, such as treatment dosage, patients’ status, and pathological stages. Secondly, the sample sizes of eight included studies are rather small and not adequate enough to detect the possible association between platinum-based chemotherapy effectiveness and ERCC1 polymorphisms. Thirdly, publication bias might be significant in our study. There are three points located at the higher-OR side and far from axles in the funnel plot (Fig. 3). It suggests the positive results are tending to be published, which may potentially influence the results of our meta-analysis.

## Conclusion

In summary, this meta-analysis suggests that the C19007T polymorphism of the ERCC1 does not contribute to the platinum-based chemotherapy effectiveness in ovarian tumor. Future large-scale studies are still needed to confirm these findings.

## Abbreviations

Asn: Asparagine
CIs: Confidence intervals
CNKI: Chinese national knowledge infrastructure
ERCC1: Excision repair cross complementation group 1
HhH2: Helix-hairpin-helix
HWE: Hardy-Weinberg equilibrium
NER: Nucleotide excision repair
OR: Odds ratios
SNPs: Single nucleotide polymorphisms
WHO: World Health Organization
